# How Autophagy Shapes the Tumor Microenvironment in Ovarian Cancer

**DOI:** 10.3389/fonc.2020.599915

**Published:** 2020-12-07

**Authors:** Alessandra Ferraresi, Carlo Girone, Andrea Esposito, Chiara Vidoni, Letizia Vallino, Eleonora Secomandi, Danny N. Dhanasekaran, Ciro Isidoro

**Affiliations:** ^1^ Laboratory of Molecular Pathology, Department of Health Sciences, Università del Piemonte Orientale “A. Avogadro”, Novara, Italy; ^2^ Stephenson Cancer Center, The University of Oklahoma Health Sciences Center, Oklahoma City, OK, United States

**Keywords:** cancer, cell metabolism, dormancy, cytokines, chemoresistance, autophagy, cancer associated fibroblasts, inflammatory stroma

## Abstract

Ovarian cancer (OC) is characterized by a high mortality rate due to the late diagnosis and the elevated metastatic potential. Autophagy, a lysosomal-driven catabolic process, contributes to the macromolecular turnover, cell homeostasis, and survival, and as such, it represents a pathway targetable for anti-cancer therapies. It is now recognized that the vascularization and the cellular composition of the tumor microenvironment influence the development and progression of OC by controlling the availability of nutrients, oxygen, growth factors, and inflammatory and immune-regulatory soluble factors that ultimately impinge on autophagy regulation in cancer cells. An increasing body of evidence indicates that OC carcinogenesis is associated, at least in the early stages, to insufficient autophagy. On the other hand, when the tumor is already established, autophagy activation provides a survival advantage to the cancer cells that face metabolic stress and protects from the macromolecules and organelles damages induced by chemo- and radiotherapy. Additionally, upregulation of autophagy may lead cancer cells to a non-proliferative dormant state that protects the cells from toxic injuries while preserving their stem-like properties. Further to complicate the picture, autophagy is deregulated also in stromal cells. Thus, changes in the tumor microenvironment reflect on the metabolic crosstalk between cancer and stromal cells impacting on their autophagy levels and, consequently, on cancer progression. Here, we present a brief overview of the role of autophagy in OC hallmarks, including tumor dormancy, chemoresistance, metastasis, and cell metabolism, with an emphasis on the bidirectional metabolic crosstalk between cancer cells and stromal cells in shaping the OC microenvironment.

## Introduction

Ovarian cancer (OC) emerges as the eighth most commonly diagnosed cancer among women worldwide and the leading cause of death among gynecological malignancies (1). Epithelial ovarian cancers are characterized by extensive genomic instability with mutations in several oncogenes and tumor suppressor genes including *BRAF, KRAS*, *TP53, BRCA1/2*, and *PTEN*, among others, that identify different subtypes with different behaviors and prognoses (2). The current therapy for OC consists in surgical debulking followed by multi-agent chemotherapy regimens with platinum-based or taxane-based compounds (3). Unfortunately, despite the initial efficacy of the treatment, the majority of OC recurs with the development of drug-resistant and metastatic tumors (3). Metastatic dissemination of OC cells predominantly occurs through direct cell spreading from the primary tumor site into the intra-abdominal cavity of OC patients that is full of malignant ascitic fluid (4). Cancer cells distribute into the cavity, broadly seed in and invade through the peritoneum, and resume secondary tumor growth in distant abdominal and pelvic organs (5). Together with chemoresistance, these phenomena not only denote the emergence of more aggressive tumor clones, but also reflect the dynamics and plasticity that occurred within the tumor microenvironment.

For a long time, cancer stroma has been considered as a passive by-stander in carcinogenesis, subjected to modification associated with the reactive inflammation. It is now recognized that the tumor stroma composition (in terms of vascular structures, cell components, and secreted factors) and its dynamic changes play a fundamental role in cancer progression (6).

Alterations in the expression profiles of genes and signaling pathways lead to aberrant stroma activation, resulting in intense extracellular matrix remodeling along with the synthesis and release of factors involved in metastasis, angiogenesis, and drug resistance (7). Overall, the stroma can either hamper or sustain the proliferation and spread of OC cells depending on its composition. Hence, targeting cancer microenvironment could be an effective therapeutic strategy to limiting OC aggressiveness and relapse, and to this end a deeper understanding of the metabolic crosstalk between OC cells and tumor stroma is crucial. In this context, autophagy, as a catabolic pathway that provides substrates for the carbohydrate, nucleotide, protein and lipid metabolisms (8), plays a pivotal role in the exchange of metabolites between cancer cells and stromal cells, thus impacting on the evolution of the tumor microenvironment.

The tumor microenvironment (TME) has been described as hypoxic, nutrient deprived, energy limited, acidic, and inflammatory (9, 10). This scenario can induce autophagy in cancer cells (11). As a further layer of complexity, autophagy in cancer and stromal cells contributes to the remodeling of the extracellular matrix and reshaping of the cellular composition in the TME (12). Since stromal cells in the TME and cancer cells influence reciprocally the regulation of autophagy in a dynamic manner that favors cancer cell persistence and progression (13, 14), interrupting this metabolic complicity could be of therapeutic value (15).

## The Ovarian Cancer Microenvironment

**Figure 1:**
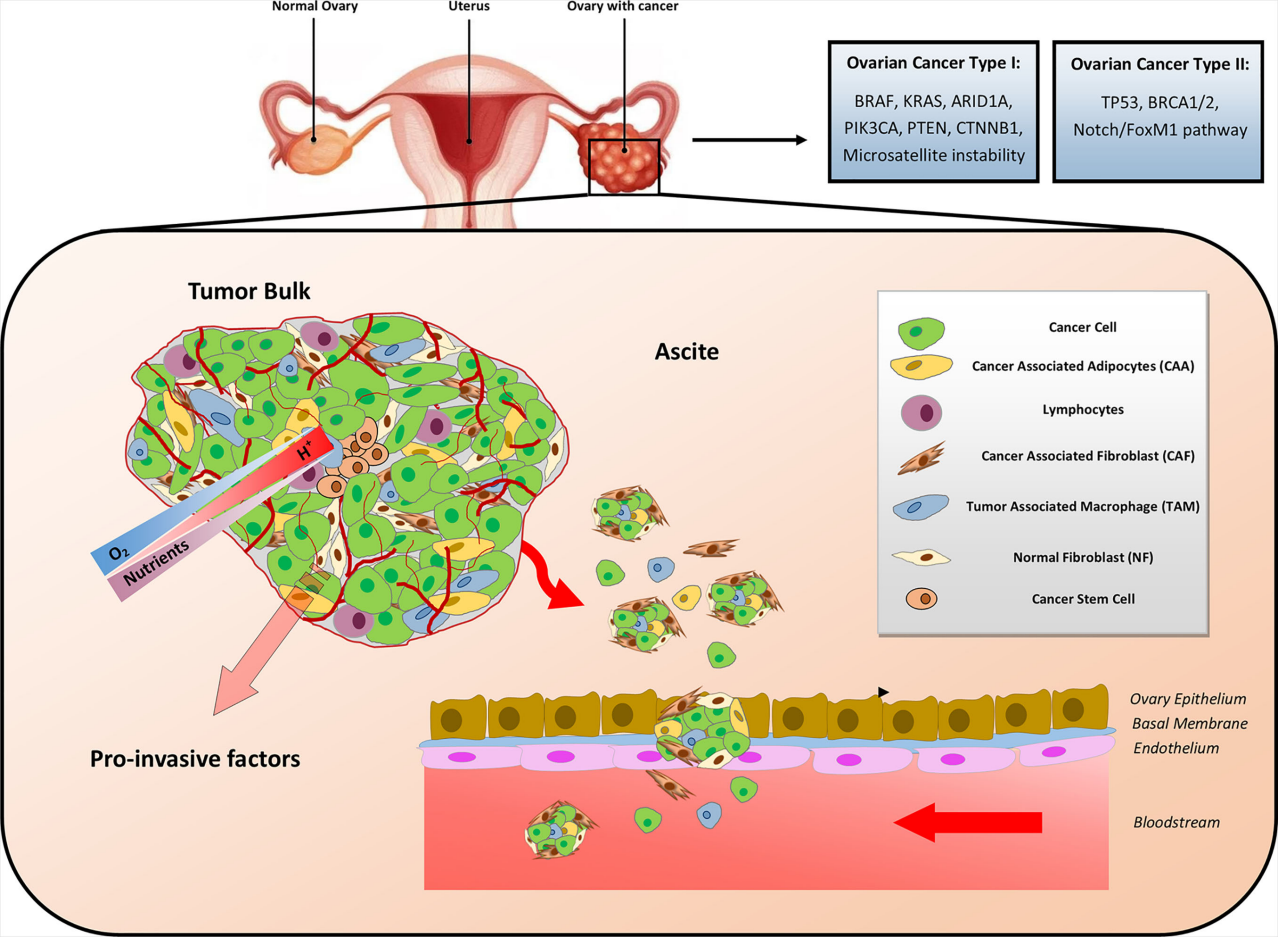
Cellular and metabolic heterogeneity of ovarian cancer microenvironment. Ovarian cancer (OC) has been classified a Type I and Type II. Type I OC (typically harboring mutations in *BRAF*, *KRAS*, *ARID1A*, *PIK3CA*, *PTEN*, and *CTNNB*1) is less aggressive and less lethal than Type II (typically harboring mutations in *TP53* and *BRCA1/2*). The metabolic rewiring occurring in the OC microenvironment is influenced by the interactions among the heterogenous cellular populations, the availability of nutrients and oxygen (O_2_), the metabolic side products, and the pH gradients. Gradients of the latter are indicated by the shaded arrows. Disseminated OC multicellular spheroids detach from the tumor bulk and spread within the abdomen, where ascite accumulation facilitates their seeding and colonization of peritoneal cavity.

Ovarian cancer is composed of organoid-like structures in which epithelial OC cells interact with the stroma composed of cancer-associated fibroblasts (CAFs), cancer-associated adipocytes (CAAs), tumor-associated macrophages (TAMs), and other immune and inflammatory cells, mesenchymal stem cells (MSCs) and endothelial cells embedded in a mixture of amorphous components forming the extracellular matrix (ECM) (16). A schematic representation of OC TME composition is illustrated in **Figure 1**.

Cellular and molecular crosstalk between stromal and cancer cells significantly affects OC tumorigenesis and progression through the establishment of a malignant *liaison* that results in a tumor-promoting and immunosuppressive microenvironment (17). In addition, the inflammatory stroma could also favor the awakening of dormant OC cells, thus causing cancer recurrence (17). Moreover, oncogene-induced inflammatory pathways facilitate metabolic changes in the tumor stroma resulting in the secretion of metabolites that are used as alternative nutrient source by cancer cells to sustain their increasing energy needs for growth and anabolic functions (18).

These bidirectional communications have a great impact on biological processes, the metabolic reprogramming of the various components of tumor stroma, and their phenotypic features *via* autocrine, paracrine, endocrine, and contact-dependent cell signaling, thus reflecting the changes driving OC relapse (19, 20).

The bioactive soluble factors secreted into the extracellular space and the malignant ascitic fluid include metabolites (amino acids, fatty acids, *etc.*), proteases (MMP1, MMP2, MMP9, uPA, collagenases), phospholipids (LPAs), chemokines (CXCL1, CXCL-11, CXCL-12, CCL5), growth factors (IGF-1, M-CSF, VEGF, HGF, FGF), and cytokines (IL-4, IL-10, IL-6, IL-1*α*, IL-1β, TGF-*β*, IL-8, TNF-α). Additionally, OC microenvironment is greatly affected not only by soluble factors, but also by epigenetic mechanisms, such as non-coding RNAs (miRNAs and lncRNAs), histone modifications, DNA methylation, and chromatin remodeling (5).

Tumor-derived metabolites accumulate in the microenvironment in consequence of an accelerated and imbalanced metabolism. Among the pathways involved in the metabolic rewiring, here we focus on autophagy, a mechanism acting as an active supplier of nutrients/energy source for cancer cell survival.

## Autophagy and Ovarian Cancer

Autophagy, literally “self-eating”, is a dynamic catabolic process that ensures cellular quality control through the degradation of cytoplasmic damaged, harmful, aged, redundant or unnecessary cellular self-constituents (11, 21).

**Figure 2:**
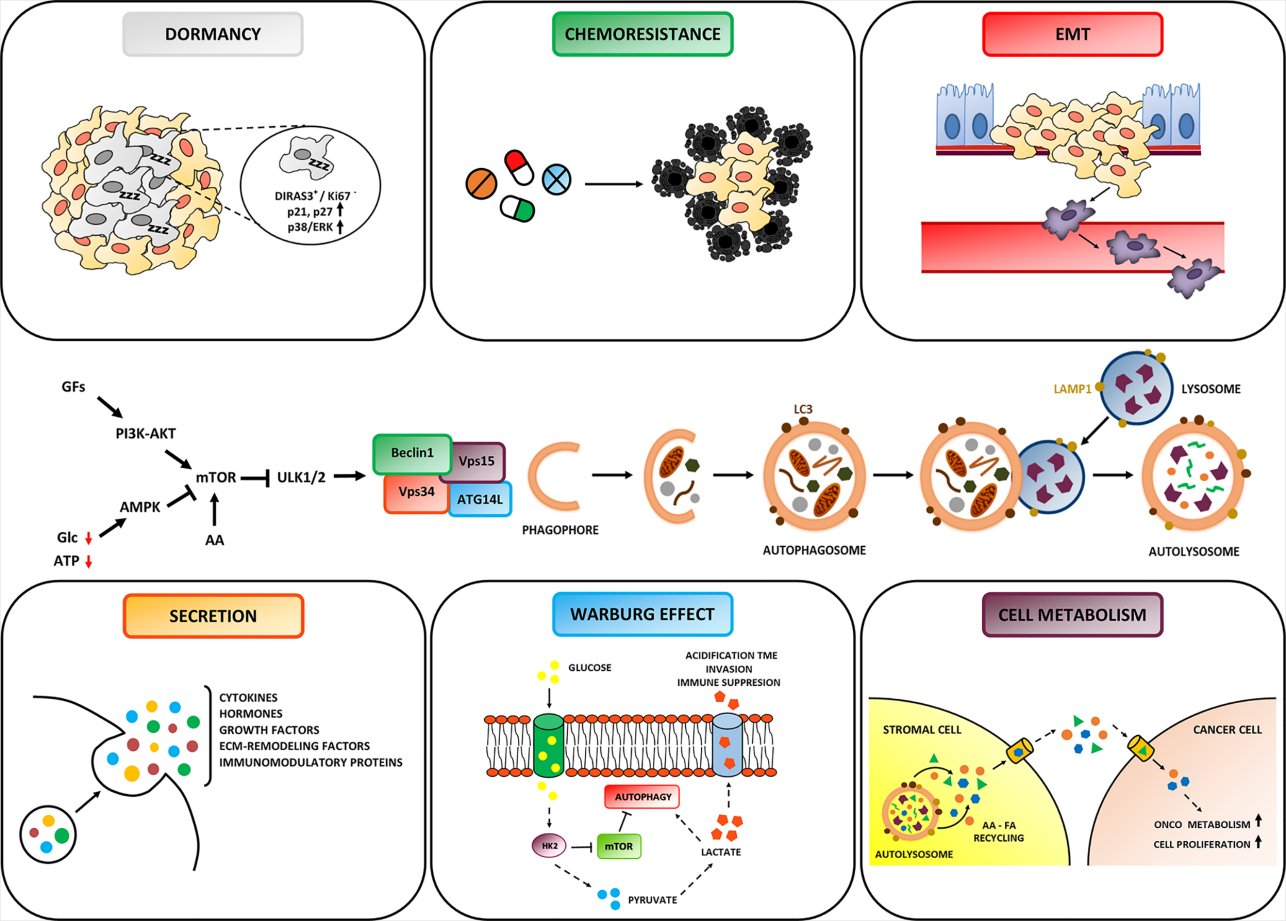
Role of Autophagy in Ovarian cancer hallmarks. Autophagy is regulated by signaling pathways that sense the presence of growth factors, the availability of nutrients (amino acids and glucose) and of ATP, and that ultimately modulate the activity of the ULK complex. The formation of the autophagosome is triggered by the BECLIN-1 interactome (comprised of Vps34, Vps15 and ATG14L). The autophagosome entraps the autophagic *cargo* and then fuses with the lysosome to form the autolysosome, wherein degradation takes place. Depending on cancer cell background and TME context, autophagy plays either an inhibitor or promoting role on cancer evolution. Particularly, autophagy can: (i) fulfill increased energetic and biosynthetic needs for tumor growth; (ii) favor resistance to cytotoxic drugs; (iii) promote the survival of dormant cells; (iv) modulate the secretion of soluble factors affecting cancer progression and anti-tumor immune response; (v) inhibit metastasis and prevent *anoikis* of invading cancer cells. Note that autophagy in stromal cells sustains the metabolic crosstalk with cancer cells.

Autophagy starts with the recruitment of key initiating complexes resulting in the nucleation of the isolation membrane, followed by the elongation of the vesicle that captures cytosolic material, forming the autophagosome. Next, the autophagosome fuses with endosomal-lysosomal organelles to form the autolysosome. The sequestered *cargo* is degraded by lysosomal acid hydrolases into monomeric constituents that are exported across the lysosomal membrane to the cytosol for re-use during cellular renovation and macromolecules turnover (22). Regarding the molecular regulation, the mTORC1 complex appears as the main negative controller of autophagy through the inhibition of the ULK1/2 complex (**Figure 2**). Both the abundant presence of amino acids, which directly activate mTOR, or of growth factors, which activate mTOR *via* the PI3KC1–AKT pathway, result in the inhibition of autophagy, while the absence of nutrients (amino acids, glucose) and the lack of oxygen, which causes a reduced production of ATP, trigger the AMPK pathway resulting in the induction of autophagy (**Figure 2**).

Under normal conditions, autophagy occurs at basal levels and ensures constitutive turnover of cytosolic components by specifically degrading damaged or redundant organelles and molecules (23). In response to a wide range of extra- and intracellular stress stimuli, autophagy is induced and sustains cell survival by producing metabolites that can be re-used in biosynthetic processes or energy production (11). It has been calculated that autophagy controls the destiny of up to fifty per cent of the cellular proteins (24), and up to four percent of the mitochondria pool is basally degraded by mitophagy every day, while dysfunctional mitochondria are actively and selectively removed on demand (25). Thus, overall autophagy presides to protein and organelle quality control that preserves cell viability and prevents from cancer (26, 27). Additional tumor suppressive mechanisms of autophagy include the elimination of oncogenic substrates and dampening of oxidative stress and inflammation, which contribute to preserve genome stability (28, 29). Yet, these same mechanisms might turn to be pro-tumorigenic when activated in established tumor cells. In fact, by removing the damaged proteins and DNA, autophagy protects cancer cells from the cytotoxic stress induced by chemo- and radiotherapy (30, 31). Further, autophagy can provide a survival advantage to the cancer cells in the most inner portion of the tumor that face hostile conditions such as hypoxic, growth factor and nutrient-deprived microenvironment due to the scarce vascularization (32). Autophagy is deregulated in OC cells in consequence of genetic and epigenetic mutations (33, 34). The haplo-insufficient tumor suppressor gene *BECN1* (that encodes for the protein BECLIN-1) has been the first autophagy gene that was found to predispose to OC development when mono-allelically deleted (35, 36). Autophagy in ovarian cancer is subjected to epigenetic regulation by histone de-acetylases (37), by hypomethylation (38), and by non-coding RNAs (39–41). Autophagy, being a stress response that can be regulated epigenetically, it is not surprising that the TME can modulate the actual level of autophagy in ovarian cancer cells (10).

## Autophagy-Mediated Metabolism Shapes the Ovarian Cancer Microenviroment

Cancer growth requires a complex and orchestrated rewiring of cell metabolism (42). Altered cell metabolism enables cancer cells to sustain their increased energetic and biosynthetic needs for tumor growth, invasion, and progression. To satisfy this abnormal high demand of metabolites the cancer cells must partner with other cells in the TME.

To this end, epithelial cancer cells can reprogram the catabolism of neighboring stromal cells to enhance their secretion of energy-rich metabolites (such as pyruvate, lactate, ketone bodies, amino acids, and free fatty acids) that are up-taken by tumor cells and used to sustain their onco-metabolism (43–45).

The unbalanced availability of these metabolites in the TME affects the regulation of autophagy which in turn has an impact on cell behavior and destiny. Below, we summarize how autophagy responds to environmental harsh conditions and how this response reflects on the composition and function of the TME and cancer cell fate. As schematically represented in **Figure 2**, autophagy is essentially implicated in the amino acid, glucose, and lipid metabolisms, in the secretion of soluble factors found in the TME, and in cancer cell behavior and fate (that includes cell dormancy, response to chemotherapeutics and metastasization).

### Autophagy in Cell Metabolism

#### Amino Acid Metabolism and Autophagy

Amino acids play a central role in cell metabolism, as they are the fundamental blocks for protein synthesis and a source for the synthesis of carbohydrates and lipids. Non-essential amino acids could be synthesized endogenously, but if the proliferation rate is fast, these amino acids have to be provided from external sources because the capacity of endogenous synthesis does not meet the increased needs of the highly proliferating cancer cells (46). Amino acid shortage not only impairs the anabolic pathways, it also potently induces autophagy for rescuing amino acids and other metabolites necessary for vital functions (47). Consistently, prolonged amino acid starvation in ovarian cancer cells triggers the expression of several autophagy genes (48). Amino acid availability is sensed by the mTOR kinase, that positively regulates protein synthesis while negatively regulates autophagy (49). In cancer cells, the lack of (conditionally) essential amino acids determines the proteasomal degradation of the mTOR kinase, thus allowing pro-survival autophagy (50). It has been shown that ovarian cancer cells cultivated in arginine-deprived medium and unable to synthesize it, preventing the induction of autophagy eventually leads to cell death (51). Cancer cells are particularly addicted to glutamine, a (conditionally) essential amino acid that fuels the mitochondrial Krebs’s cycle, particularly when glucose is not fully oxidized in the mitochondria (52). In cancer cells, there is a cooperative link between the glutamine and the glucose metabolisms (53).

Nutrient addiction displayed by OC cells relies on the availability of different substrates, and these metabolic dependencies are also dependent on cancer stage (44, 54, 55).

Interestingly, low-invasive OC cells are basically glutamine-independent and rely on glucose metabolism for their growth, whereas high-invasive OC cells shift their nutrient addiction from glucose to glutamine (56). Remarkably, to fulfill their increased requirement for glutamine, highly malignant OC cells reprogram their surrounding CAFs to neo-synthetize more glutamine than what normal ovarian fibroblasts would do (56). Glutamine has been shown to stimulate OC cell proliferation through modulation of the mTOR pathway (57), and this likely inhibits autophagy as indicated by the observation that L-asparaginase, which degrades glutamine to glutamate, induces autophagy in OC cells (58).

#### Glucose Metabolism and Autophagy

Glucose is one of the principal sources of energy, and its lack is sensed by the cell through the Hexokinase 2-mTOR axis to trigger autophagy as a stress response (59). Cancer cells resident in well-vascularized areas can fully exploit glucose through mitochondrial respiration to sustain their energetic needs (60). However, highly proliferating cancer cells preferably use glycolysis to produce ATP and skip the mitochondrial respiration step despite the availability of oxygen, a phenomenon known as Warburg effect (61). On the other hand, cancer cells located in hypoxic niches are obliged to use glycolysis for the production of ATP because of the lack of oxygen (62). Glycolysis is ten times less convenient than mitochondrial respiration in terms of ATP yield, therefore the tumor mass consumes huge amount of glucose (approximately 10–200 times more) compared to the normal counterpart tissue, and this leads to the production of high amount of lactate (63).

The acidification of the TME due to lactate secretion further contributes to cancer progression promoting (i) cancer cell invasion and metastasis (64), (ii) Epithelial-to-Mesenchymal transition (EMT) and the emergence of stem-like clones (65, 66), (iii) angiogenesis (67), (iv) the survival of cancer cells competing with stromal and immune cells for energy and glucose availability (68, 69), (v) the polarization of tumor-associated macrophages (TAMs) toward an immune suppressive phenotype (70, 71), and (vi) resistance to hypoxia, cytotoxic drugs and immune attack (72).

It has been proposed that the glycolytic shift in glucose metabolism along with upregulation of autophagy occur also in stromal cells, mainly in CAFs (44), that would then supply the cancer cells with amino acids and lactate to sustain the anabolic processes involved in hyperproliferation and cancer progression (73, 74). In the presence of oxygen, cancer cells can oxidize lactate into pyruvate thanks to the lactate dehydrogenase B (LDHB), thus substituting the glucose to fuel the mitochondria (75). Interestingly, this LDHB-dependent metabolism increases the autophagy flux along with increased acidification of the lysosomes in cancer cells (75).

#### Fatty Acid Metabolism and Autophagy

The bidirectional crosstalk between ovarian cancer cells and adipocytes, that are used as “food donors”, is another example of metabolic complicity in the TME that supports OC growth (76, 77).

Lipidomic analysis of malignant OC ascites revealed the presence of high concentrations of linoleic acid, a potent PPAR*β/δ* agonist that promotes the pro-tumorigenic polarization of ovarian TAMs (78).

When cultured in the ascite microenvironment, ovarian cancer cells undergo a metabolic shift from aerobic glycolysis to *β*-oxidation and lipogenesis, and this associates with increased aggressiveness (79).

In the TME, cancer-associated adipocytes (CAAs) release fatty acids (FAs) that are used by cancer cells as an energetic source, and adipokines that stimulate cancer progression (80). Interestingly, the uptake of (long chain) FAs present in the TME is higher in hypoxic cancer cells due to HIF-1*α*-induced expression of CD36, also known as FA translocase (81). To be noted, CD36 is over expressed in ovarian cancer cells and associates with ovarian cancer progression and metastasis (82). In hepatocarcinoma cells, over-expression of CD36 has been shown not only to increase the long-chain FA uptake but also to inhibit autophagy, and particularly lipophagy (83). Whether this also occurs in OC cells remains to be demonstrated.

Again, recent evidences suggest that lipophagy, the autophagic degradation of lipids, has a role in the release of free fatty acids that constitute an alternative source of energy as well as metabolic intermediates involved in cell signaling and macromolecule biosynthesis (84).

During energy stress condition, CAAs upregulate autophagy and supply fatty acids that are transported to cancer cells and can be used for building cell membranes or are catabolized by *β*-oxidation (85). The latter process may also generate ketone bodies that represent a useful substrate for ATP generation in fast-growing cancer cells, since ketone bodies provide more ATP with less oxygen consumption than glucose (86). This aspect leads to speculation that cancer cells growing in an adipocyte-enriched microenvironment, like OC, specifically rely on mitochondrial *β*-oxidation to fuel high bioenergetic demand of cancer cells, suggesting that adipocytes promote a metabolic switch to favor fatty acids over glucose and amino acids as mitochondrial substrate due to a tissue-specific feature of this cancer (80, 87).

In particular settings, lipophagy has shown to exert a tumor-suppressive role. The lack of lysosomal acid lipases, the enzymes involved in lipophagic degradation, results in the promotion of cancer growth and metastasis through the mTOR-dependent activation of myeloid-derived suppressor cells (88).

Further characterization of lipophagy perturbation in the TME is needed to fully understand its role in OC development and progression and to determine its potential as a target for novel cancer therapeutic approaches (89).

### The Role of Secretory Autophagy in Shaping the TME

Peptides, proteins, and hormones that lack the leader/secretion peptide and fail to enter the conventional secretory system, normally operating *via* the endoplasmic reticulum–Golgi pathway, can be secreted in an autophagy-dependent manner (90). Thus, autophagy actively participates in remodeling of TME *via* unconventional secretion of soluble factors involved in intercellular communications (91). Autophagy knockdown in stromal and/or in cancer cells results in the reduction of cytokine and chemokine release (including IL-1β, IL-18, IL-6, IL-8, IL-2, CCL2, CCL20, TNFα, LIF), indicating an autophagy-dependent secretion of pro-inflammatory and pro-invasive factors that collectively modulate tumor growth, immune evasion, stemness maintenance, angiogenesis, and metastasis (91–97). Additionally, the autophagy-dependent tumor secretome includes extracellular matrix remodeling proteins (*e.g.* MMP2, MMP9), angiogenic stimuli (VEGFA) and growth factors (b-FGF, TGF*β*1) (95, 98, 99). Further, autophagy impairment hampers the secretion of IFN-*γ*, CXCL9, CXCL10, CXCL11 that are involved in T cells and dendritic cell recruitment, resulting in immune surveillance escape (100).

On the other hand, autophagy promotes the release of immunomodulatory proteins known as DAMPs (damage-associated molecular patterns) that elicit anti-tumor immunity by activating immune cells, thus limiting tumor progression (101, 102).

## Autophagy-Mediated Crosstalk Within the Microenvironment Affects Ovarian Cancer Progression

The TME has been described as inflammatory and desmoplastic, recalling a wound that never heals (103). In normal tissues subjected to environmental injuries, the inflammatory and autophagy pathways cross-communicate and cooperate to re-establish homeostasis (104). In the tumor niche, several pro-inflammatory factors and pro-tumorigenic metabolites could directly or indirectly impinge on autophagy in cancer and stromal cells, thus affecting cancer evolution (105, 106). On the other side, autophagy plays a role in the release of pro-tumorigenic cytokines (107). IL-6, one such inflammatory and pro-tumorigenic cytokine released by CAFs, is particularly secreted in the OC TME and accumulates abundantly in the ascitic fluid of OC (17). This cytokine has been shown to promote EMT and the migration of OC cells (108). Recently, we have shown that the pro-EMT and pro-migratory effect of IL-6 on OC cells is dependent on the epigenetic regulation of autophagy (40).

Another molecule highly secreted within the OC microenvironment is lysophosphatidic acid (LPA). In ovarian cancer, LPA could act either in an autocrine or a paracrine manner to reprogram the glucose metabolism (109), to induce EMT and invasiveness (110), and to promote the secretion of pro-invasive factors (like VEGF, IL-6, IL-8, *etc.*) (111, 112). It is likely that these effects are mediated by modulation of autophagy. Accordingly, LPA has been shown to inhibit autophagy in prostate cancer cells (113, 114). Further, it has been demonstrated that LPA induces a glycolytic shift that associates with the pheno-conversion of ovarian normal fibroblasts into ovarian CAFs (115), a process that depends on autophagy modulation (74).

Consistent with a role of autophagy in metastatic dissemination, a recent study found that disseminated ovarian cancer cells within the peritoneum over-expressed several autophagy genes (116). However, the relationship between EMT and autophagy is quite intricate: while on one hand it inhibits the early phases of metastasization mainly through the selective degradation of EMT-associated transcription factors such as SNAIL, SLUG, and TWIST, on the other hand it helps cancer cells that already underwent EMT to prevent from *anoikis* and move within the ECM (117), yet it is also involved in the reverse Mesenchymal-to-Epithelial transition process (118, 119).

Tumor dormancy coincides with the period during which disseminated tumor cells can remain in a latent state associated with inhibition of cell proliferation and reprogramming of cell metabolism that sustain cell survival with the least expenditure of energy in presence of reduced source of nutrients and oxygen (120). Hence, dormancy is an important adaptive response to microenvironmental stresses, and it is the result of an integrated metabolic crosstalk (involving cytokines, chemokines, growth factors, metabolites, and non-coding RNAs) in which autophagy plays a key role (121, 122). Dormancy and autophagy are functionally interconnected at the molecular level. The tumor suppressor gene *ARH-I* (also known as *DIRAS3*), which is epigenetically silenced in a vast majority of ovarian cancers, promotes autophagy in cultured OC cells (40, 123), and its inactivation in the context of a xenografted tumor in mice causes the interruption of dormancy and the rapid re-growth of the tumor (124).

This mechanism of adaptation to a nutrient poor microenvironment favors the survival of chemoresistant OC cells (125). Therefore, autophagy signaling machinery might integrate quiescence and survival signals to promote damage repair, *via* ATG7 regulation of p53 and sustain cancer cell needs by generating an alternative route for amino acid turnover as well as for energetic metabolism and ATP balance (126, 127).

## Conclusions

Here we have briefly described the interplay occurring within the OC tumor microenvironment between OC cells and stromal components, how this influences the dynamic changes in the structure and composition of extracellular matrix and of tumor stroma, the reprogramming of energetic metabolism, and the secretion of soluble factors that, overall, negatively impact on the prognosis of OC patients.

Deregulation of autophagy has been recognized as one of the main hallmarks of cancer. However, while studying the role of autophagy in cancer, we now realize that things are far more complicated than what it was thought some decades ago. In fact, we must consider that autophagy regulation is subjected both to genetic and epigenetic mutations of oncogenes and tumor suppressor genes, and it is transitorily modulated by microenvironmental factors such as metabolites, pro-inflammatory cytokines and pro-oxidant molecules. As a further layer of complexity, we have to consider the level of autophagy in both the epithelial cancer cells, the stromal cells (CAFs, CAAs, TAMs), and probably also of neighboring normal cells, and keep in mind that all these actors can exchange metabolites and factors influencing reciprocally the autophagy level. Interrupting the functional link between autophagy and cell metabolism both in epithelial cancer cells and in stromal cells could be an effective therapeutic approach to slow down OC progression and prevent relapse. To achieve this goal, we need to further investigate the mechanisms of autophagy dynamics and how it shapes the TME.

## Author Contributions

AF, CG, AE, and CV: drafted the manuscript and iconography. LV and ES: literature search and iconography. DD and CI: conceptualized, supervised, and finalized the manuscript. All authors contributed to the article and approved the submitted version.

## Funding

AF is a recipient of a post-doctoral fellowship “Paolina Troiano” (id. 24094) granted by Associazione Italiana per la Ricerca sul Cancro (AIRC, Milan, Italy). LV and ES are PhD students recipient of a fellowship granted by the Italian Ministry of Education, University and Research (MIUR, Rome, Italy) with the contribution of Associazione per la Ricerca Medica Ippocrate-Rhazi (ARM-IR, Novara, Italy). CV was supported with a fellowship from Associazione per la Ricerca Medica Ippocrate-Rhazi (ARM-IR, Novara, Italy).

## Conflict of Interest

The authors declare that the research was conducted in the absence of any commercial or financial relationships that could be construed as a potential conflict of interest.

## Abbreviations

OC, ovarian cancer; miRNA, microRNA, lncRNA, long non-coding RNA; EMT, Epithelial-to-Mesenchymal transition; CAF, cancer-associated fibroblast; CAA, cancer-associated adipocyte; TAM, tumor-associated macrophage; MSC, mesenchymal stem cell; TME, tumor microenvironment; ECM, extracellular matrix; AAs, amino acids; FAs, fatty acids.

## References

[1] Cabasag CJ Arnold M Butler J Inoue M Trabert B Webb PM The influence of birth cohort and calendar period on global trends in ovarian cancer incidence. Int J Cancer (2020) 146(3):749–58. 10.1002/ijc.32322 PMC6786921 30968402

[2] Cook DP Vanderhyden BC Ovarian cancer and the evolution of subtype classifications using transcriptional profiling. Biol Reprod (2019) 101(3):645–58. 10.1093/biolre/ioz099 31187121

[3] Coleman RL Monk BJ Sood AK Herzog TJ Latest research and treatment of advanced-stage epithelial ovarian cancer. Nat Rev Clin Oncol (2013) 10(4):211–24. 10.1038/nrclinonc.2013.5 PMC3786558 23381004

[4] Kipps E Tan DS Kaye SB Meeting the challenge of ascites in ovarian cancer: new avenues for therapy and research. Nat Rev Cancer (2013) 13(4):273–82. 10.1038/nrc3432 PMC4673904 23426401

[5] Klymenko Y Nephew KP Epigenetic Crosstalk between the Tumor Microenvironment and Ovarian Cancer Cells: A Therapeutic Road Less Traveled. Cancers (Basel) (2018) 10(9):pii: E295. 10.3390/cancers10090295 PMC6162502 30200265

[6] Langley RR Fidler IJ The seed and soil hypothesis revisited–the role of tumor-stroma interactions in metastasis to different organs. Int J Cancer (2011) 128(11):2527–35. 10.1002/ijc.26031 PMC3075088 21365651

[7] Lili LN Matyunina LV Walker LD Benigno BB McDonald JF Molecular profiling predicts the existence of two functionally distinct classes of ovarian cancer stroma. BioMed Res Int (2013) 2013:846387. 10.1155/2013/846387 23762861 PMC3665167

[8] Guo JY White E Autophagy, Metabolism, and Cancer. Cold Spring Harb Symp Quant Biol (2016) 81:73–8. 10.1101/sqb.2016.81.030981 PMC5521269 28209717

[9] Wojtkowiak JW Rothberg JM Kumar V Schramm KJ Haller E Proemsey JB Chronic autophagy is a cellular adaptation to tumor acidic pH microenvironments. Cancer Res (2012) 72(16):3938–47. 10.1158/0008-5472.CAN-11-3881 PMC3749826 22719070

[10] Yang X Yu DD Yan F Jing YY Han ZP Sun K The role of autophagy induced by tumor microenvironment in different cells and stages of cancer. Cell Biosci (2015) 5:14. 10.1186/s13578-015-0005-2. eCollection 2015. 25844158 PMC4384293

[11] Kroemer G Mariño G Levine B Autophagy and the integrated stress response. Mol Cell (2010) 40(2):280–93. 10.1016/j.molcel.2010.09.023 PMC3127250 20965422

[12] Maes H Rubio N Garg AD Agostinis P Autophagy: shaping the tumor microenvironment and therapeutic response. Trends Mol Med (2013) 19(7):428–46. 10.1016/j.molmed.2013.04.005 23714574

[13] Folkerts H Hilgendorf S Vellenga E Bremer E Wiersma VR The multifaceted role of autophagy in cancer and the microenvironment. Med Res Rev (2019) 39(2):517–60. 10.1002/med.21531 PMC6585651 30302772

[14] Ngabire D Kim GD Autophagy and Inflammatory Response in the Tumor Microenvironment. Int J Mol Sci (2017) 18(9):2016. 10.3390/ijms18092016 PMC5618664 28930154

[15] Jiang Y Wang C Zhou S Targeting tumor microenvironment in ovarian cancer: Premise and promise. Biochim Biophys Acta Rev Cancer (2020) 1873(2):188361. 10.1016/j.bbcan.2020.188361 32234508

[16] Worzfeld T Pogge von Strandmann E Huber M Adhikary T Wagner U Reinartz S The Unique Molecular and Cellular Microenvironment of Ovarian Cancer. Front Oncol (2017) 7:24. 10.3389/fonc.2017.00024. eCollection 2017. Review. 28275576 PMC5319992

[17] Thuwajit C Ferraresi A Titone R Thuwajit P Isidoro C The metabolic cross-talk between epithelial cancer cells and stromal fibroblasts in ovarian cancer progression: Autophagy plays a role. Med Res Rev (2018) 38(4):1235–54. 10.1002/med.21473 PMC6032948 28926101

[18] Ahmed N Escalona R Leung D Chan E Kannourakis G Tumour microenvironment and metabolic plasticity in cancer and cancer stem cells: Perspectives on metabolic and immune regulatory signatures in chemoresistant ovarian cancer stem cells. Semin Cancer Biol (2018) 53:265–81. 10.1016/j.semcancer.2018.10.002 30317036

[19] Nowak M Glowacka E Szpakowski M Szyllo K Malinowski A Kulig A Proinflammatory and immunosuppressive serum, ascites and cyst fluid cytokines in patients with early and advanced ovarian cancer and benign ovarian tumors. Neuro Endocrinol Lett (2010) 31(3):375–83. 20588232

[20] Rainczuk A Rao J Gathercole J Stephens AN The emerging role of CXC chemokines in epithelial ovarian cancer. Reproduction (2012) 144(3):303–17. 10.1530/REP-12-0153 22771929

[21] Yang Z Klionsky DJ Eaten alive: a history of macroautophagy. Nat Cell Biol (2010) 12(9):814–22. 10.1038/ncb0910-814 PMC3616322 20811353

[22] Kuma A Mizushima N Physiological role of autophagy as an intracellular recycling system: with an emphasis on nutrient metabolism. Semin Cell Dev Biol (2010) 21(7):683–90. 10.1016/j.semcdb.2010.03.002 20223289

[23] Parzych KR Klionsky DJ An overview of autophagy: morphology, mechanism, and regulation. Antioxid Redox Signal (2014) 20(3):460–73. 10.1089/ars.2013.5371 PMC3894687 23725295

[24] Mathew R Khor S Hackett SR Rabinowitz JD Perlman DH White E Functional role of autophagy-mediated proteome remodeling in cell survival signaling and innate immunity. Mol Cell (2014) 55(6):916–30. 10.1016/j.molcel.2014.07.019 PMC4169768 25175026

[25] Ma K Chen G Li W Kepp O Zhu Y Chen Q Mitophagy, Mitochondrial Homeostasis, and Cell Fate. Front Cell Dev Biol (2020) 8:467. 10.3389/fcell.2020.00467 32671064 PMC7326955

[26] Amaravadi RK Kimmelman AC Debnath J Targeting Autophagy in Cancer: Recent Advances and Future Directions. Cancer Discovery (2019) 9(9):1167–81. 10.1158/2159-8290.CD-19-0292 PMC7306856 31434711

[27] Wang Y Liu HH Cao YT Zhang LL Huang F Yi C The Role of Mitochondrial Dynamics and Mitophagy in Carcinogenesis, Metastasis and Therapy. Front Cell Dev Biol (2020) 8:413. 10.3389/fcell.2020.00413 32587855 PMC7297908

[28] White E Deconvoluting the context-dependent role for autophagy in cancer. Nat Rev Cancer (2012) 12(6):401–10. 10.1038/nrc3262 PMC3664381 22534666

[29] Singh SS Vats S Chia AY Tan TZ Deng S Ong MS Dual role of autophagy in hallmarks of cancer. Oncogene (2018) 37(9):1142–58. 10.1038/s41388-017-0046-6 29255248

[30] Ho CJ Gorski SM Molecular Mechanisms Underlying Autophagy-Mediated Treatment Resistance in Cancer. Cancers (Basel) (2019) 11(11):1775. 10.3390/cancers11111775 PMC6896088 31717997

[31] Pérez-Hernández M Arias A Martínez-García D Pérez-Tomás R Quesada R Soto-Cerrato V Targeting Autophagy for Cancer Treatment and Tumor Chemosensitization. Cancers (Basel) (2019) 11(10):1599. 10.3390/cancers11101599 PMC6826429 31635099

[32] Goldsmith J Levine B Debnath J Autophagy and cancer metabolism. Methods Enzymol (2014) 542:25–57. 10.1016/B978-0-12-416618-9.00002-9 24862259 PMC5839656

[33] Peracchio C Alabiso O Valente G Isidoro C Involvement of autophagy in ovarian cancer: a working hypothesis. J Ovarian Res (2012) 5(1):22. 10.1186/1757-2215-5-22 22974323 PMC3506510

[34] Zhan L Zhang Y Wang W Song E Fan Y Li J Autophagy as an emerging therapy target for ovarian carcinoma. Oncotarget (2016) Dec 13 7(50):83476–87. 10.18632/oncotarget.13080 PMC5347782 27825125

[35] Qu X Yu J Bhagat G Furuya N Hibshoosh H Troxel A Promotion of tumorigenesis by heterozygous disruption of the beclin 1 autophagy gene. J Clin Invest (2003) 112(12):1809–20. 10.1172/JCI20039 PMC297002 14638851

[36] Delaney JR Patel CB Bapat J Jones CM Ramos-Zapatero M Ortell KK Autophagy gene haploinsufficiency drives chromosome instability, increases migration, and promotes early ovarian tumors. PloS Genet (2020) 16(1):e1008558. 10.1371/journal.pgen.1008558 31923184 PMC6953790

[37] Lapinska K Housman G Byler S Heerboth S Willbanks A Oza A The Effects of Histone Deacetylase Inhibitor and Calpain Inhibitor Combination Therapies on Ovarian Cancer Cells. Anticancer Res (2016) 36(11):5731–42. 10.21873/anticanres.11156 27793894

[38] Liao YP Chen LY Huang RL Su PH Chan MW Chang CC Hypomethylation signature of tumor-initiating cells predicts poor prognosis of ovarian cancer patients. Hum Mol Genet (2014) 23(7):1894–906. 10.1093/hmg/ddt583 PMC3943526 24256813

[39] Titone R Morani F Follo C Vidoni C Mezzanzanica D Isidoro C Epigenetic control of autophagy by microRNAs in ovarian cancer. BioMed Res Int (2014) 2014:343542. 10.1155/2014/343542 24877083 PMC4022060

[40] Ferraresi A Phadngam S Morani F Galetto A Alabiso O Chiorino G Resveratrol inhibits IL-6-induced ovarian cancer cell migration through epigenetic up-regulation of autophagy. Mol Carcinog (2017) 56(3):1164–81. 10.1002/mc.22582 27787915

[41] Vallino L Ferraresi A Vidoni C Secomandi E Esposito A Dhanasekaran DN Modulation of non-coding RNAs by resveratrol in ovarian cancer cells: *In silico* analysis and literature review of the anti-cancer pathways involved. J Tradit Complement Med (2020) 10(3):217–29. 10.1016/j.jtcme.2020.02.006 PMC7340874 32670816

[42] Still ER Yuneva MO Hopefully devoted to Q: targeting glutamine addiction in cancer. Br J Cancer (2017) 116(11):1375–81. 10.1038/bjc.2017.113 PMC5520092 28441384

[43] Pavlides S Whitaker-Menezes D Castello-Cros R Flomenberg N Witkiewicz AK Frank PG The reverse Warburg effect: aerobic glycolysis in cancer associated fibroblasts and the tumor stroma. Cell Cycle (2009) 8(23):3984–4001. 10.4161/cc.8.23.10238 19923890

[44] Lisanti MP Martinez-Outschoorn UE Sotgia F Oncogenes induce the cancer-associated fibroblast phenotype: metabolic symbiosis and “fibroblast addiction” are new therapeutic targets for drug discovery. Cell Cycle (2013) 12(17):2723–32. 10.4161/cc.25695 PMC3899185 23860382

[45] Fiaschi T Marini A Giannoni E Taddei ML Gandellini P De Donatis A Reciprocal metabolic reprogramming through lactate shuttle coordinately influences tumor-stroma interplay. Cancer Res (2012) 72(19):5130–40. 10.1158/0008-5472.CAN-12-1949 22850421

[46] Bhutia YD Babu E Ramachandran S Ganapathy V Amino Acid transporters in cancer and their relevance to “glutamine addiction”: novel targets for the design of a new class of anticancer drugs. Cancer Res (2015) 75(9):1782–8. 10.1158/0008-5472.CAN-14-3745 25855379

[47] Kaur J Debnath J Autophagy at the crossroads of catabolism and anabolism. Nat Rev Mol Cell Biol (2015) 16(8):461–72. 10.1038/nrm4024 26177004

[48] Ferraresi A Titone R Follo C Castiglioni A Chiorino G Dhanasekaran DN The protein restriction mimetic Resveratrol is an autophagy inducer stronger than amino acid starvation in ovarian cancer cells. Mol Carcinog (2017) 56(12):2681–91. 10.1002/mc.22711 28856729

[49] Meijer AJ Lorin S Blommaart EF Codogno P Regulation of autophagy by amino acids and MTOR-dependent signal transduction. Amino Acids (2015) 47(10):2037–63. 10.1007/s00726-014-1765-4 PMC4580722 24880909

[50] Follo C Vidoni C Morani F Ferraresi A Seca C Isidoro C Amino acid response by Halofuginone in Cancer cells triggers autophagy through proteasome degradation of mTOR. Cell Commun Signal (2019) 17(1):39. 10.1186/s12964-019-0354-2 31046771 PMC6498594

[51] Shuvayeva G Bobak Y Igumentseva N Titone R Morani F Stasyk O Single amino acid arginine deprivation triggers prosurvival autophagic response in ovarian carcinoma SKOV3. BioMed Res Int (2014) 2014:505041. 10.1155/2014/505041 24987688 PMC4058691

[52] Scalise M Pochini L Galluccio M Console L Indiveri C Glutamine Transport and Mitochondrial Metabolism in Cancer Cell Growth. Front Oncol (2017) 7:306. 10.3389/fonc.2017.00306 29376023 PMC5770653

[53] Unterlass JE Curtin NJ Warburg and Krebs and related effects in cancer. Expert Rev Mol Med (2019) 21:e4. 10.1017/erm.2019.4 31558177

[54] Benjamin DI Cravatt BF Nomura DK Global profiling strategies for mapping dysregulated metabolic pathways in cancer. Cell Metab (2012) 16(5):565–77. 10.1016/j.cmet.2012.09.013 PMC3539740 23063552

[55] Caneba CA Bellance N Yang L Pabst L Nagrath D Pyruvate uptake is increased in highly invasive ovarian cancer cells under anoikis conditions for anaplerosis, mitochondrial function, and migration. Am J Physiol Endocrinol Metab (2012) 303(8):E1036. 10.1152/ajpendo.00151.2012 22895781

[56] Yang L Moss T Mangala LS Marini J Zhao H Wahlig S Metabolic shifts toward glutamine regulate tumor growth, invasion and bioenergetics in ovarian cancer. Mol Syst Biol (2014) 10:728. 10.1002/msb.20134892 24799285 PMC4188042

[57] Yuan L Sheng X Willson AK Roque DR Stine JE Guo H Glutamine promotes ovarian cancer cell proliferation through the mTOR/S6 pathway. Endocr Relat Cancer (2015) 22(4):577–91. 10.1530/ERC-15-0192 PMC4500469 26045471

[58] Furusawa A Miyamoto M Takano M Tsuda H Song YS Aoki D Ovarian cancer therapeutic potential of glutamine depletion based on GS expression. Carcinogenesis (2018) 39(6):758–66. 10.1093/carcin/bgy033 29617730

[59] Roberts DJ Tan-Sah VP Ding EY Smith JM Miyamoto S Hexokinase-II positively regulates glucose starvation-induced autophagy through TORC1 inhibition. Mol Cell (2014) 53(4):521–33. 10.1016/j.molcel.2013.12.019 PMC3943874 24462113

[60] Porporato PE Dhup S Dadhich RK Copetti T Sonveaux P Anticancer targets in the glycolytic metabolism of tumors: a comprehensive review. Front Pharmacol (2011) 2:49. 10.3389/fphar.2011.00049 21904528 PMC3161244

[61] Liberti MV Locasale JW The Warburg Effect: How Does it Benefit Cancer Cells? Trends Biochem Sci (2016) 41(3):211–8. 10.1016/j.tibs.2015.12.001. Epub 2016 Jan 5. Erratum in: Trends Biochem Sci. 2016 Mar;41(3):287. Erratum in: Trends Biochem Sci. 2016 Mar;41(3):287. PMID: 26778478; PMCID: PMC4783224. PMC4783224 26778478

[62] Moreno-Sánchez R Rodríguez-Enríquez S Marín-Hernández A Saavedra E Energy metabolism in tumor cells. FEBS J (2007) 274(6):1393–418. 10.1111/j.1742-4658.2007.05686.x 17302740

[63] Vander Heiden MG Cantley LC Thompson CB Understanding the Warburg effect: the metabolic requirements of cell proliferation. Science (2009) 324(5930):1029–33. 10.1126/science.1160809 PMC2849637 19460998

[64] Estrella V Chen T Lloyd M Wojtkowiak J Cornnell HH Ibrahim-Hashim A Acidity generated by the tumor microenvironment drives local invasion. Cancer Res (2013) 73(5):1524–35. 10.1158/0008-5472.CAN-12-2796 PMC3594450 23288510

[65] Varum S Rodrigues AS Moura MB Momcilovic O Easley CA4 Ramalho-Santos J Energy metabolism in human pluripotent stem cells and their differentiated counterparts. PloS One (2011) 6(6):e20914. 10.1371/journal.pone.0020914 21698063 PMC3117868

[66] Xu Q Zhang Q Ishida Y Hajjar S Tang X Shi H EGF induces epithelial-mesenchymal transition and cancer stem-like cell properties in human oral cancer cells via promoting Warburg effect. Oncotarget (2017) 8(6):9557–71. 10.18632/oncotarget.13771 PMC5354753 27926487

[67] Dhup S Dadhich RK Porporato PE Sonveaux P Multiple biological activities of lactic acid in cancer: influences on tumor growth, angiogenesis and metastasis. Curr Pharm Des (2012) 18(10):1319–30. 10.2174/138161212799504902 22360558

[68] Ho PC Bihuniak JD Macintyre AN Staron M Liu X Amezquita R Phosphoenolpyruvate Is a Metabolic Checkpoint of Anti-tumor T Cell Responses. Cell (2015) 162(6):1217–28. 10.1016/j.cell.2015.08.012 PMC4567953 26321681

[69] Chang CH Qiu J O’Sullivan D Buck MD Noguchi T Curtis JD Metabolic Competition in the Tumor Microenvironment Is a Driver of Cancer Progression. Cell (2015) 162(6):1229–41. 10.1016/j.cell.2015.08.016 PMC4864363 26321679

[70] Colegio OR Chu NQ Szabo AL Chu T Rhebergen AM Jairam V Functional polarization of tumour-associated macrophages by tumour-derived lactic acid. Nature (2014) 513(7519):559–63. 10.1038/nature13490 PMC4301845 25043024

[71] Icard P Kafara P Steyaert JM Schwartz L Lincet H The metabolic cooperation between cells in solid cancer tumors. Biochim Biophys Acta (2014) 1846(1):216–25. 10.1016/j.bbcan.2014.06.002 24983675

[72] Icard P Shulman S Farhat D Steyaert JM Alifano M Lincet H How the Warburg effect supports aggressiveness and drug resistance of cancer cells? Drug Resist Updat (2018) 38:1–11. 10.1016/j.drup.2018.03.001 29857814

[73] Carito V Bonuccelli G Martinez-Outschoorn UE Whitaker-Menezes D Caroleo MC Cione E Metabolic remodeling of the tumor microenvironment: migration stimulating factor (MSF) reprograms myofibroblasts toward lactate production, fueling anabolic tumor growth. Cell Cycle (2012) 11(18):3403–14. 10.4161/cc.21701 PMC3466551 22918248

[74] Yan Y Chen X Wang X Zhao Z Hu W Zeng S The effects and the mechanisms of autophagy on the cancer-associated fibroblasts in cancer. J Exp Clin Cancer Res (2019) 38(1):171. 10.1186/s13046-019-1172-5 31014370 PMC6480893

[75] Brisson L Bański P Sboarina M Dethier C Danhier P Fontenille MJ Lactate Dehydrogenase B Controls Lysosome Activity and Autophagy in Cancer. Cancer Cell (2016) 30(3):418–31. 10.1016/j.ccell.2016.08.005 27622334

[76] Nieman KM Kenny HA Penicka CV Ladanyi A Buell-Gutbrod R Zillhardt MR Adipocytes promote ovarian cancer metastasis and provide energy for rapid tumor growth. Nat Med (2011) 17(11):1498–503. 10.1038/nm.2492 PMC4157349 22037646

[77] Motohara T Masuda K Morotti M Zheng Y El-Sahhar S Chong KY An evolving story of the metastatic voyage of ovarian cancer cells: cellular and molecular orchestration of the adipose-rich metastatic microenvironment. Oncogene (2019) 38(16):2885–98. 10.1038/s41388-018-0637-x PMC6755962 30568223

[78] Schumann T Adhikary T Wortmann A Finkernagel F Lieber S Schnitzer E Deregulation of PPARβ/δ target genes in tumor-associated macrophages by fatty acid ligands in the ovarian cancer microenvironment. Oncotarget (2015) 6(15):13416–33. 10.18632/oncotarget.3826 PMC4537024 25968567

[79] Chen RR Yung MMH Xuan Y Zhan S Leung LL Liang RR Targeting of lipid metabolism with a metabolic inhibitor cocktail eradicates peritoneal metastases in ovarian cancer cells. Commun Biol (2019) 2:281. 10.1038/s42003-019-0508-1 31372520 PMC6668395

[80] Nieman KM Romero IL Van Houten B Lengyel E Adipose tissue and adipocytes support tumorigenesis and metastasis. Biochim Biophys Acta (2013) 1831(10):1533–41. 10.1016/j.bbalip.2013.02.010 PMC3742583 23500888

[81] Lengyel E Makowski L DiGiovanni J Kolonin MG Cancer as a Matter of Fat: The Crosstalk between Adipose Tissue and Tumors. Trends Cancer (2018) 4(5):374–84. 10.1016/j.trecan.2018.03.004 PMC5932630 29709261

[82] Ladanyi A Mukherjee A Kenny HA Johnson A Mitra AK Sundaresan S Adipocyte-induced CD36 expression drives ovarian cancer progression and metastasis. Oncogene (2018) 37(17):2285–301. 10.1038/s41388-017-0093-z PMC5920730 29398710

[83] Li Y Yang P Zhao L Chen Y Zhang X Zeng S CD36 plays a negative role in the regulation of lipophagy in hepatocytes through an AMPK-dependent pathway. J Lipid Res (2019) 60(4):844–55. 10.1194/jlr.M090969 PMC6446711 30662007

[84] Maan M Peters JM Dutta M Patterson AD Lipid metabolism and lipophagy in cancer. Biochem Biophys Res Commun (2018) 504(3):582–9. 10.1016/j.bbrc.2018.02.097 PMC6086774 29438712

[85] Wen YA Xing X Harris JW Zaytseva YY Mitov MI Napier DL Adipocytes activate mitochondrial fatty acid oxidation and autophagy to promote tumor growth in colon cancer. Cell Death Dis (2017) 8(2):e2593. 10.1038/cddis.2017.21 28151470 PMC5386470

[86] Seyfried TN Flores RE Poff AM D’Agostino DP Cancer as a metabolic disease: implications for novel therapeutics. Carcinogenesis (2014) 35(3):515–27. 10.1093/carcin/bgt480 PMC3941741 24343361

[87] Lyssiotis CA Kimmelman AC Metabolic Interactions in the Tumor Microenvironment. Trends Cell Biol (2017) 27(11):863–75. 10.1016/j.tcb.2017.06.003 PMC5814137 28734735

[88] Zhao T Du H Ding X Walls K Yan C Activation of mTOR pathway in myeloid-derived suppressor cells stimulates cancer cell proliferation and metastasis in lal(-/-) mice. Oncogene (2015) 34(15):1938–48. 10.1038/onc.2014.143 PMC4254377 24882582

[89] Kounakis K Chaniotakis M Markaki M Tavernarakis N Emerging Roles of Lipophagy in Health and Disease. Front Cell Dev Biol (2019) 7:185. 10.3389/fcell.2019.00185 31552248 PMC6746960

[90] Keulers TG Schaaf MB Rouschop KM Autophagy-Dependent Secretion: Contribution to Tumor Progression. Front Oncol (2016) 6:251. 10.3389/fonc.2016.00251 27933272 PMC5122571

[91] Ponpuak M Mandell MA Kimura T Chauhan S Cleyrat C Deretic V Secretory autophagy. Curr Opin Cell Biol (2015) 35:106–16. 10.1016/j.ceb.2015.04.016 PMC4529791 25988755

[92] Dupont N Jiang S Pilli M Ornatowski W Bhattacharya D Deretic V Autophagy-based unconventional secretory pathway for extracellular delivery of IL-1β. EMBO J (2011) 30(23):4701–11. 10.1038/emboj.2011.398 PMC3243609 22068051

[93] Maycotte P Jones KL Goodall ML Thorburn J Thorburn A Autophagy Supports Breast Cancer Stem Cell Maintenance by Regulating IL6 Secretion. Mol Cancer Res (2015) 13(4):651–8. 10.1158/1541-7786.MCR-14-0487 PMC4398616 25573951

[94] Salah FS Ebbinghaus M Muley VY Zhou Z Al-Saadi KR Pacyna-Gengelbach M Tumor suppression in mice lacking GABARAP, an Atg8/LC3 family member implicated in autophagy, is associated with alterations in cytokine secretion and cell death. Cell Death Dis (2016) 7(4):e2205. 10.1038/cddis.2016.93 27124579 PMC4855672

[95] Zhan Z Xie X Cao H Zhou X Zhang XD Fan H Autophagy facilitates TLR4- and TLR3-triggered migration and invasion of lung cancer cells through the promotion of TRAF6 ubiquitination. Autophagy (2014) 10(2):257–68. 10.4161/auto.27162 PMC5396095 24321786

[96] Kraya AA Piao S Xu X Zhang G Herlyn M Gimotty P Identification of secreted proteins that reflect autophagy dynamics within tumor cells. Autophagy (2015) 11(1):60–74. 10.4161/15548627.2014.984273 25484078 PMC4502670

[97] Cotzomi-Ortega I Aguilar-Alonso P Reyes-Leyva J Maycotte P Autophagy and Its Role in Protein Secretion: Implications for Cancer Therapy. Mediators Inflamm (2018) 2018:4231591. 10.1155/2018/4231591 30622432 PMC6304875

[98] New J Arnold L Ananth M Alvi S Thornton M Werner L Secretory Autophagy in Cancer-Associated Fibroblasts Promotes Head and Neck Cancer Progression and Offers a Novel Therapeutic Target. Cancer Res (2017) 77(23):6679–91. 10.1158/0008-5472.CAN-17-1077 PMC5712244 28972076

[99] Nüchel J Ghatak S Zuk AV Illerhaus A Mörgelin M Schönborn K TGFB1 is secreted through an unconventional pathway dependent on the autophagic machinery and cytoskeletal regulators. Autophagy (2018) 14(3):465–86. 10.1080/15548627.2017.1422850 PMC5915026 29297744

[100] Wei H Wei S Gan B Peng X Zou W Guan JL Suppression of autophagy by FIP200 deletion inhibits mammary tumorigenesis. Genes Dev (2011) 25(14):1510–27. 10.1101/gad.2051011 PMC3143941 21764854

[101] Martins I Wang Y Michaud M Ma Y Sukkurwala AQ Shen S Molecular mechanisms of ATP secretion during immunogenic cell death. Cell Death Differ (2014) 21(1):79–91. 10.1038/cdd.2013.75 23852373 PMC3857631

[102] Michaud M Martins I Sukkurwala AQ Adjemian S Ma Y Pellegatti P Autophagy-dependent anticancer immune responses induced by chemotherapeutic agents in mice. Science (2011) 334(6062):1573–7. 10.1126/science.1208347 22174255

[103] Dvorak HF Tumors: wounds that do not heal-redux. Cancer Immunol Res (2015) 3(1):1–11. 10.1158/2326-6066.CIR-14-0209 25568067 PMC4288010

[104] Cadwell K Crosstalk between autophagy and inflammatory signalling pathways: balancing defence and homeostasis. Nat Rev Immunol (2016) 16(11):661–75. 10.1038/nri.2016.100 PMC5343289 27694913

[105] Martinez-Outschoorn UE Whitaker-Menezes D Lin Z Flomenberg N Howell A Pestell RG Cytokine production and inflammation drive autophagy in the tumor microenvironment: role of stromal caveolin-1 as a key regulator. Cell Cycle (2011) 10(11):1784–93. 10.4161/cc.10.11.15674 PMC3142462 21566463

[106] Monkkonen T Debnath J Inflammatory signaling cascades and autophagy in cancer. Autophagy (2018) 14(2):190–8. 10.1080/15548627.2017.1345412 PMC5902219 28813180

[107] Thongchot S Ferraresi A Vidoni C Loilome W Yongvanit P Namwat N Resveratrol interrupts the pro-invasive communication between cancer associated fibroblasts and cholangiocarcinoma cells. Cancer Lett (2018) 430:160–71. 10.1016/j.canlet.2018.05.031 29802929

[108] Wang Y Li L Guo X Jin X Sun W Zhang X Interleukin-6 signaling regulates anchorage-independent growth, proliferation, adhesion and invasion in human ovarian cancer cells. Cytokine (2012) 59(2):228–36. 10.1016/j.cyto.2012.04.020 22595649

[109] Ha JH Radhakrishnan R Jayaraman M Yan M Ward JD Fung KM LPA Induces Metabolic Reprogramming in Ovarian Cancer via a Pseudohypoxic Response. Cancer Res (2018) 78(8):1923–34. 10.1158/0008-5472.CAN-17-1624 PMC5899640 29386184

[110] Ha JH Ward JD Radhakrishnan R Jayaraman M Song YS Dhanasekaran DN Lysophosphatidic acid stimulates epithelial to mesenchymal transition marker Slug/Snail2 in ovarian cancer cells via Gαi2, Src, and HIF1α signaling nexus. Oncotarget (2016) 7(25):37664–79. 10.18632/oncotarget.9224 PMC5122340 27166196

[111] Jeon ES Heo SC Lee IH Choi YJ Park JH Choi KU Ovarian cancer-derived lysophosphatidic acid stimulates secretion of VEGF and stromal cell-derived factor-1 alpha from human mesenchymal stem cells. Exp Mol Med (2010) 42(4):280–93. 10.3858/emm.2010.42.4.027 PMC2859327 20177148

[112] Fang X Yu S Bast RC Liu S Xu HJ Hu SX Mechanisms for lysophosphatidic acid-induced cytokine production in ovarian cancer cells. J Biol Chem (2004) 279(10):9653–61. 10.1074/jbc.M306662200 14670967

[113] Chang CL Liao JJ Huang WP Lee H Lysophosphatidic acid inhibits serum deprivation-induced autophagy in human prostate cancer PC-3 cells. Autophagy (2007) 3(3):268–70. 10.4161/auto.3909 17329959

[114] Genc GE Hipolito VEB Botelho RJ Gumuslu S Lysophosphatidic acid represses autophagy in prostate carcinoma cells. Biochem Cell Biol (2018) 97(4):387–96. 10.1139/bcb-2018-0164 30403494

[115] Radhakrishnan R Ha JH Jayaraman M Liu J Moxley KM Isidoro C Ovarian cancer cell-derived lysophosphatidic acid induces glycolytic shift and cancer-associated fibroblast-phenotype in normal and peritumoral fibroblasts. Cancer Lett (2019) 442:464–74. 10.1016/j.canlet.2018.11.023 PMC7780843 30503552

[116] Kuo CL Jiang ZY Wang YW Lin TY Huang WL Wu FJ In vivo selection reveals autophagy promotes adaptation of metastatic ovarian cancer cells to abdominal microenvironment. Cancer Sci (2019) 110(10):3204–14. 10.1111/cas.14162 PMC6778661 31385416

[117] Gugnoni M Sancisi V Manzotti G Gandolfi G Ciarrocchi A Autophagy and epithelial-mesenchymal transition: an intricate interplay in cancer. Cell Death Dis (2016) 7(12):e2520. 10.1038/cddis.2016.415 27929542 PMC5260980

[118] Catalano M D’Alessandro G Lepore F Corazzari M Caldarola S Valacca C Autophagy induction impairs migration and invasion by reversing EMT in glioblastoma cells. Mol Oncol (2015) 9(8):1612–25. 10.1016/j.molonc.2015.04.016 PMC5528793 26022108

[119] Qiang L He YY Autophagy deficiency stabilizes TWIST1 to promote epithelial-mesenchymal transition. Autophagy (2014) 10(10):1864–5. 10.4161/auto.32171 PMC4198370 25126736

[120] Vera-Ramirez L Hunter KW Tumor cell dormancy as an adaptive cell stress response mechanism. F1000Res (2017) 6:2134. 10.12688/f1000research.12174.1 29263786 PMC5730860

[121] Aguirre-Ghiso JA Models, mechanisms and clinical evidence for cancer dormancy. Nat Rev Cancer (2007) 7(11):834–46. 10.1038/nrc2256 PMC2519109 17957189

[122] Gao XL Zhang M Tang YL Liang XH Cancer cell dormancy: mechanisms and implications of cancer recurrence and metastasis. Onco Targets Ther (2017) 10:5219–28. 10.2147/OTT.S140854. eCollection 2017. Review. PMC5667781 29138574

[123] Lu Z Baquero MT Yang H Yang M Reger AS Kim C DIRAS3 regulates the autophagosome initiation complex in dormant ovarian cancer cells. Autophagy (2014) J 10(6):1071–92. 10.4161/auto.28577. Erratum in: Autophagy. 2014 Aug;10(8):1482. PubMed PMID: 24879154; PubMed Central PMCID: PMC4091169. PMC4091169 24879154

[124] Lu Z Luo RZ Lu Y Zhang X Yu Q Khare S The tumor suppressor gene ARHI regulates autophagy and tumor dormancy in human ovarian cancer cells. J Clin Invest (2008) 118(12):3917–29. 10.1172/JCI35512 PMC2582930 19033662

[125] Sutton MN Huang GY Zhou J Mao W Langley R Lu Z Amino Acid Deprivation-Induced Autophagy Requires Upregulation of DIRAS3 through Reduction of E2F1 and E2F4 Transcriptional Repression. Cancers (Basel) (2019) 11(5):pii: E603. 10.3390/cancers11050603 PMC6562629 31052266

[126] Lee IH Kawai Y Fergusson MM Rovira II Bishop AJ Motoyama N Atg7 modulates p53 activity to regulate cell cycle and survival during metabolic stress. Science (2012) 336(6078):225–8. 10.1126/science.1218395 PMC4721513 22499945

[127] Liang J Shao SH Xu ZX Hennessy B Ding Z Larrea M The energy sensing LKB1-AMPK pathway regulates p27(kip1) phosphorylation mediating the decision to enter autophagy or apoptosis. Nat Cell Biol (2007) 9(2):218–24. 10.1038/ncb1537 17237771

